# Application of a simple skin stretching system and negative pressure wound therapy in repair of complex diabetic foot wounds

**DOI:** 10.1186/s13018-021-02405-6

**Published:** 2021-04-14

**Authors:** Yaojun Wu, Liang Chen, Shaokun Wu, Liying Yu, Mimi Chen, Jingnan Wang, Jiejie Chen, Qingjiang Pang

**Affiliations:** Department of Orthopedics, Hwa Mei Hospital, University of Chinese Academy of Sciences (Ningbo No. 2 Hospital), No. 41 Northwest Street, Ningbo, 315010 Zhejiang China

**Keywords:** Simple skin stretching system, Negative pressure wound therapy, Diabetic foot wounds

## Abstract

The management of complex diabetic foot wounds with large skin defects poses a challenge for surgeons. We presented a simple skin stretching system and negative pressure wound therapy for the repair of complex diabetic foot wounds to examine the effectiveness and safety.

A total of 16 patients with diabetic foot ulcers were retrospectively reviewed between January 2015 and October 2020. All patients underwent the treatment by 3 stages. In stage 2, these difficult-to-close wounds of diabetes foot were residual. This method was applied to the wounds with a median defect size of 20.42 cm^2^ (range, 4.71–66.76 cm^2^).

The median time for closure of complex diabetic foot wounds was 14 days ranging from 8 to 19 days. With respect to the absolute rates of reduction, it was observed with a median of 1.86 cm^2^/day, ranging from 0.29 cm^2^/day to 8.35 cm^2^/day. In accordance with the localization of the defect, the patients were divided into 3 groups: side of the foot (37.5%), dorsum of the foot (50.0%), and others (12.5%). There was no statistical difference between side of the foot and dorsum of the foot in terms of the median defect size with *P* = 0.069 (Kruskal–Wallis test). Otherwise, there were statistically significant differences regarding the median time and the median absolute rates (*P* < 0.05; Kruskal–Wallis test). No severe complications were encountered in this study.

In summary, our results show that application of the simple skin stretching system and NPWT is an effective and safe approach to complex diabetic foot wounds. Nevertheless, more attention should be paid to the appropriate patient selection and intraoperative judgment to ensure wound closure and avoid undue complications.

## Introduction

Diabetes mellitus (DM) is a severe and complex disease with significant socioeconomic and health care implications, both in developed and developing nations. There were more than 415 million adults with DM in 2017, and the prevalence of diabetes is estimated to get 642 million by 2040 all over the world [1]. Specifically, the published literatures have shown that the estimated prevalence of adult diabetes is 11.6% and the prevalence of prediabetes is 50.1%, which means there are 113.9 million with diabetes and 493.4 million with prediabetes in China [2].

Unfortunately, the prevalence of diabetic foot ulcers (DFUs) is up to 15% among the patients with DM [3]. This is secondary to a variety of diabetes-related risk factors, such as peripheral neuropathy and vascular disease [4]. Diabetic foot ulcers (DFUs) are associated with a high rate of hospitalization and a 20-fold increased risk of lower limb amputations [5]. Various DFU treatments have been reported in the literatures, including off-loading, debridement, revascularization, negative pressure wound therapy (NPWT), growth factors, platelet-rich plasma, and skin graft [6–10]. NPWT has proved to be an effective method of accelerating the healing of DFUs. However, for complex diabetic foot wounds with large skin defects that cannot be stitched together by low tension, wound reduction is limited with application of NPWT alone. In order to solve the problem of large skin defects, many skin stretching devices have been brought in. All of them are designed to reduce local mechanical loads on skin and take advantage of the skin mechanical properties of creep and stress relaxation for the wound closure [11].

Thus, we present this retrospective observational study by application of a simple skin stretching system and NPWT in repair of complex diabetic foot wounds. We hypothesized that the method would be effective and safe for wound healing.

## Patients and methods

### Patients

In our single-center study, 16 patients with DFUs were retrospectively reviewed between January 2015 and October 2020 in the Department of Orthopedics, Hwa Mei Hospital, University of Chinese Academy of Sciences (Ningbo No. 2 Hospital), Ningbo, Zhejiang, China. Patients were included in the study if their complex diabetic foot wounds that cannot be primarily closed were residual after debridement. The exclusion criteria included poor skin viscoelasticity, renal failure, poor compliance with medical treatments, and ischemic ulcer that needed revascularization. The study protocol was approved by the ethics committee of Hwa Mei hospital and performed in accordance with the ethical standards prescribed by the Helsinki Declaration. Informed consent was obtained from all the participants before the treatment in the study.

### Methods

All patients underwent the treatment by 3 stages. In stage 1, the patient’s wound was sharply debrided and digitally photographed as soon as possible. During the surgery, infected and nonviable soft tissues were absolutely debrided. The decision to excise the infected bone was made, combined with the preoperative radiographs and intraoperative finding. The debridement was finished when the skin, soft tissue, and bone visually appeared normal. Then, the NPWT system (VSD Medical Science and Technology Co. Ltd., Wuhan, China) was applied to the diabetic wound that could not be primarily closed and it could provide controlled negative pressures between 50 and 100 mmHg. The NPWT dressing was changed 3–5 days once after the initial operation until the diabetic foot wound had no clinical signs and symptoms of infection.

In stage 2, these difficult-to-close wounds of diabetic foot were residual. Determining which large defects of the wounds were suitable for the simple skin stretching system, including assessing the wound size as well as the mobility and quality of the neighboring cutaneous condition, was all-important. If the viscoelastic properties of skin were good, staples were placed along the margins of the diabetic foot wound at 1.5 to 2-cm intervals. Subsequently, one elastic band derived from 7# surgical glove was inserted through the staples, and crossed over the whole wound. The elastic band was tied with knots at both ends to sustain gentle but constant uniform tension at each anchoring point along the margins of the wound. Finally, the wound was connected with the NPWT system as described before. This method created a gradual wound closure by persist traction on the skin margins, which could be periodically applied until definitive wound closure was achieved.

In stage 3, the tension of the wound closure was assessed according to the intraoperative findings. During the procedure of the simple skin stretching system, the skin tension was reduced over time. If the tension was low, the wounds could be sutured finally.

### Statistical analysis

Data collected for this study included basic demographics, duration and severity of DFU, wound length and width, area of the defect (length × width × pi/4), time to definite closure (from stage 2 to wound closure finally), and complications by 2 independent wound care specialist nurses. A visual analogue scale (VAS) was used to assess the pain, which ranged from 0, no pain, to 10, most intense pain.

Statistical analysis of the data was performed using the Statistical Package for the Social Sciences (SPSS), version 20.0 (SPSS, Inc., Chicago, IL, USA). All quantitative variables were expressed as means and standard deviation (SD) and skewed data were written by median values and range. Kruskal–Wallis test was used to compare groups when appropriate. The results were considered statistically significant at the level of *P* < 0.05 in all applied analyses.

## Results

This retrospective observational clinical study analyzed the outcome of 16 patients with complex diabetic foot wounds residual. There were 12 male and 4 female patients. The mean age of all patients was 60.6 ± 10.5 years with a mean follow-up of 10.8 ± 3.1 months (Table 1). All of the patients were type 2 DM, and the mean duration of diabetes was 9.6 ± 4.8 years. The diabetic foot ulcers were divided by Wagner classification with 3 cases (18.8%) of Grade 3 and 13 cases (81.2%) of Grade 4. There were 6 patients who were hypertensive, 4 patients with ischemic heart disease, 14 patients with peripheral neuropathy, 7 patients with nephropathy, and 8 patients with retinopathy.

**Table 1:** Baseline demographics and clinical characteristics of the study population

Variable	Total, *n* = 16
Age (mean ± SD, years)	60.6 ± 10.5
Gender	
Male	12 (75%)
Female	4 (25%)
Type 2 DM	16 (100%)
Duration of diabetes (mean ± SD, years)	9.6 ± 4.8
Wagner classification	
Grade 3	3 (18.8%)
Grade 4	13 (81.2%)
Follow-up (mean ± SD, months)	10.8 ± 3.1
Comorbidities	
Hypertension	6 (37.5%)
Ischemic heart disease	4 (25.0%)
Peripheral neuropathy	14 (87.5%)
Nephropathy	7 (43.8%)
Retinopathy	8 (50.0%)

After the treatment of stage 1, these complex diabetic foot wounds were residual. The defect size of complex diabetic foot wounds ranged from 4.71 to 66.76 cm^2^ with a median defect size of 20.42 cm^2^ (Table 2). According to the localization of the defect, the patients were divided into 3 groups: side of the foot (37.5%), dorsum of the foot (50.0%), and others (12.5%) (Table 2). The median time for complex diabetic foot wound closure was 14 days ranging from 8 days to 19 days (Table 2). With respect to the absolute rates of reduction, it was observed with a median of 1.86 cm^2^/day, ranging from 0.29 cm^2^/day to 8.35 cm^2^/day.

**Table 2:** Wound-related study characteristics and outcomes. For quantitative variables, median and range are calculated

Variable	Total, *n* = 16
Wound location	
Bilateral side of the foot	6 (37.5%)
Dorsum of the foot	8 (50.0%)
Others	2 (12.5%)
Wound size (cm^2^)	20.42 (4.71–66.76)
Time for wound closure (days)	14 (8–19)
Absolute reduction (cm^2^/day)	1.86 (0.29–8.35)

**Fig. 1:**
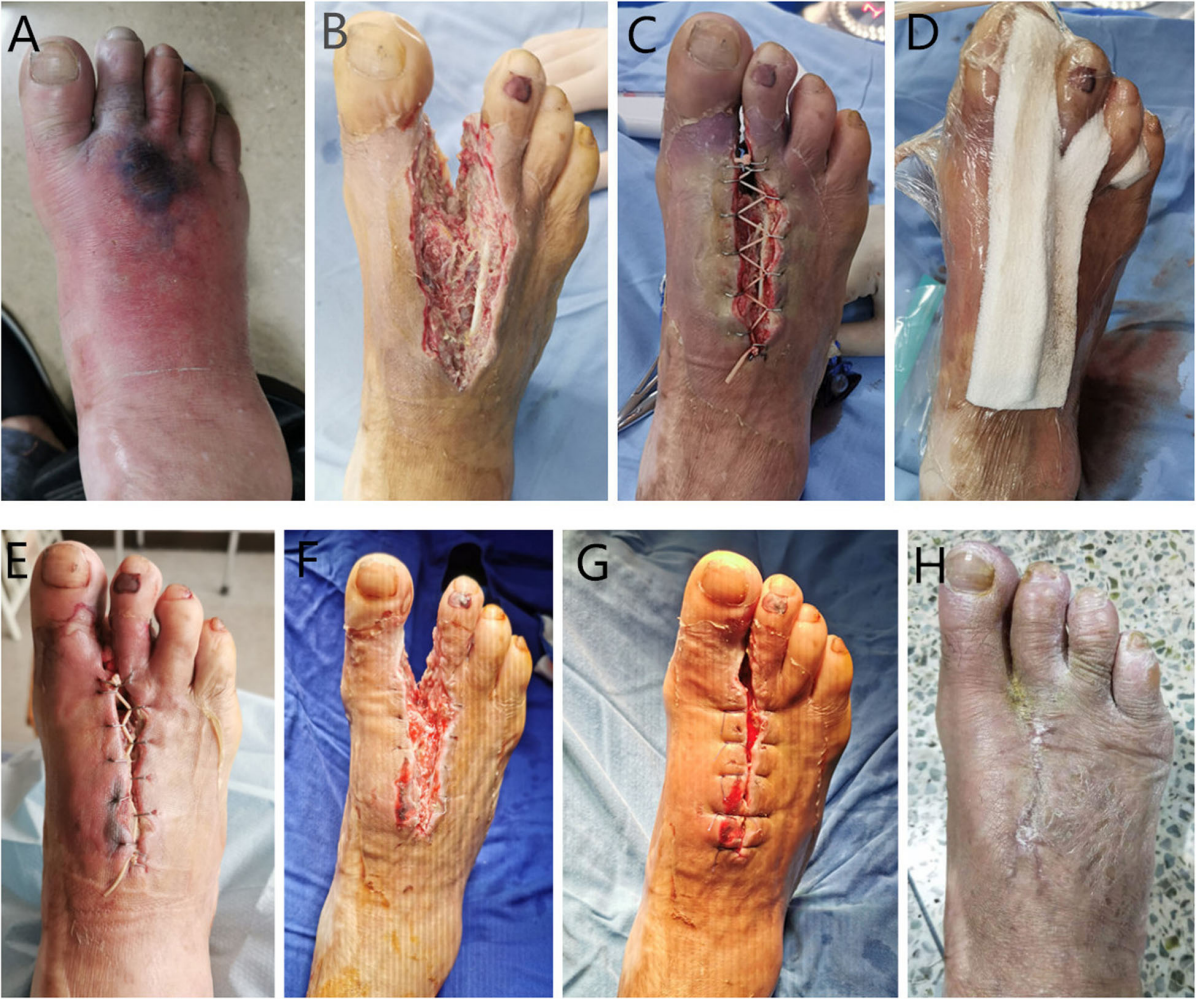
54-year-old man, diabetic foot, Wagner classification: grade 4. **a** Diabetic foot condition with second toe necrosis after admission. **b** Diabetic foot condition after absolute debridement. **c** Application of the simple skin stretching system and condition of the margin of the wound. **d** Use NPWT system simultaneously. **e** Diabetic wound condition after removing the NPWT system. **f** The condition of the wound after removing the staples and elastic band. **g** The wound was sutured without high tension. **h** 3 months after last operation

**Fig. 2:**
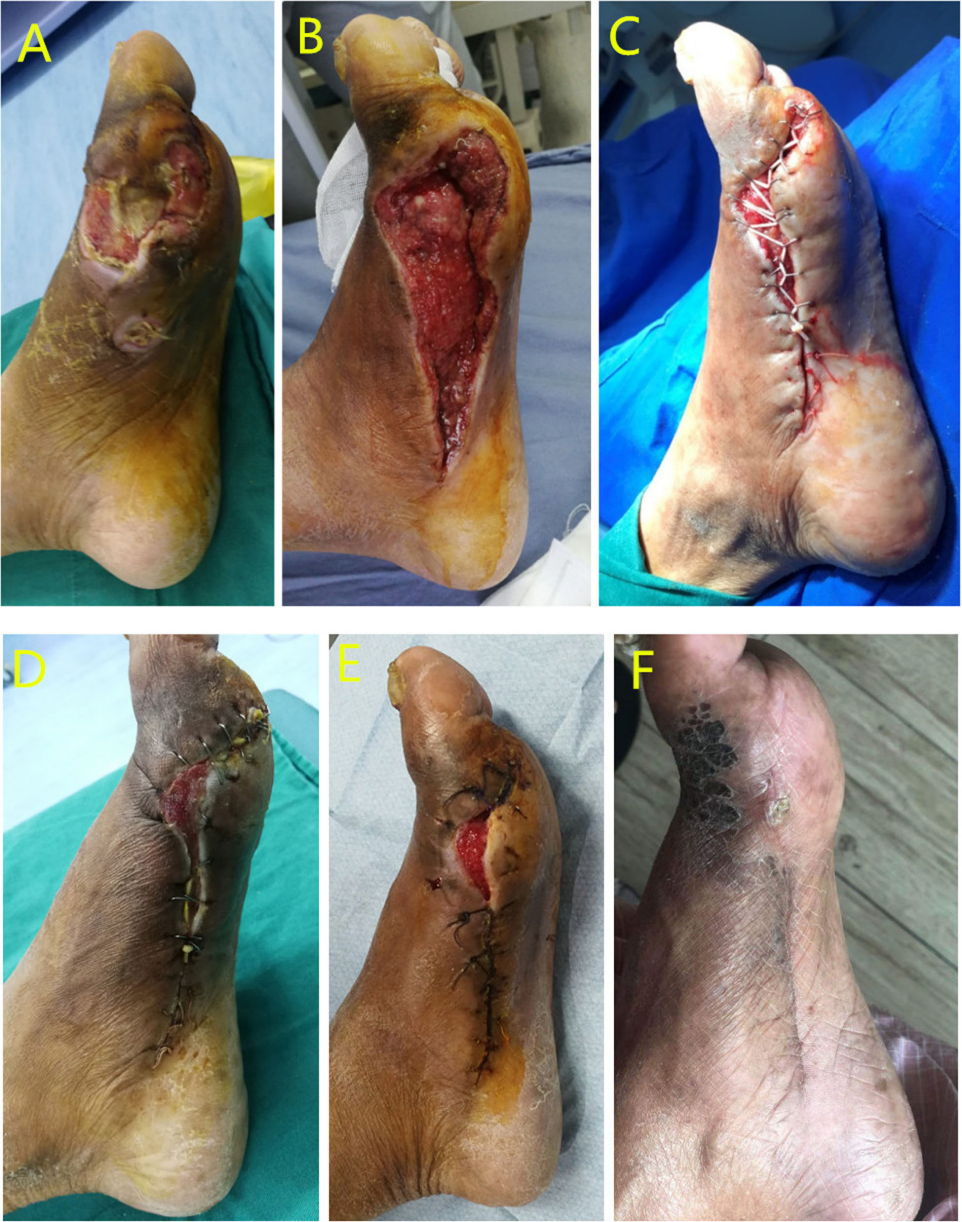
75-year-old man, diabetic foot ulcer, Wagner classification: grade 3. **a** Diabetic foot condition on the tibial side of the foot after admission. **b** Diabetic foot condition after debridement. **c** Application of the simple skin stretching system. **d** Diabetic wound condition after removing the NPWT system. **e** The staples and elastic band were removed, and the wound was sutured. **f** 3 months after last operation

The median wound size observed on the side of the foot was 26.69 cm^2^ (range, 16.49–66.76 cm^2^), which was 18.85 cm^2^ (range, 14.13–25.13 cm^2^) on the dorsum of the foot (Table 3). There was no statistically difference between 2 groups in terms of the median defect size with *P* = 0.069 (Kruskal–Wallis test). The median time for diabetic foot wound closure was 10.5 days ranging from 8 days to 14 days on the side of the foot (Table 3). Defects on the dorsum of the foot were closed within a median time of 14.5 days (range, 10–19 days). The median time between 2 groups was statistically significant different with *P* < 0.05 (Kruskal–Wallis test). The absolute rates of wound reduction were observed on the side of the foot with a median of 2.32 cm^2^/day (range, 2.06–8.35 cm^2^/day) followed by the dorsum of the foot with 1.22 cm^2^/day (range, 0.99–2.09 cm^2^/day). There were statistically significant differences between 2 groups in the absolute rates of wound reduction (*P* < 0.05; Kruskal–Wallis test), thus it became obvious that different anatomical areas on the foot had different viscoelastic properties (Table 3). Figures 1 and 2 were examples of the progression of wound healing using the simple skin stretching system and NPWT.

**Table 3:** Surgical data separated by localization (*n* = 14). For quantitative variables, median and range as well as the *P*-value arising from Kruskal–Wallis test are calculated

	Bilateral side of the foot	Dorsum of the foot	*P*-value
Wound size (cm^2^)	26.69 (16.49–66.76)	18.85 (14.13–25.13)	*P* = 0.069
Time for wound closure (days)	10.5 (8–14)	14.5 (10–19)	*P* < 0.05
Absolute reduction (cm^2^/day)	2.32 (2.06–8.35)	1.22 (0.99–2.09)	*P* < 0.05

All the defects were finally closed without skin graft or local flaps, and the scars were relatively acceptable. No severe complications were encountered in this study, such as amputation and death. Some staples were dislodged due to point loading from tightening or full range of motion in 2 cases. Three cases of skin edge necrosis observed were healed after several wound dressings changed. Retrospectively, patients felt just a little pain during the procedure of the method. Pain relief was achieved through oral analgesics.

## Discussion

We presented the results of this retrospective observation of patients with diabetic foot ulcers (DFUs) and large skin defects residual after debridement. The main findings supported our hypothesis that application of the simple skin stretching system and negative pressure wound therapy in repair of complex diabetic foot wound was effective and safe.

Negative pressure wound therapy (NPWT) reported by Argenta and Morykwas [12] is generally used all over the world. NPWT reduces extracellular edema, improves local blood flow, and stimulates local angiogenesis to increase the formation of granulation tissue [13–15]. In addition, wound bioburden and risk of infection are decreased, and wound healing is accelerated [16, 17]. Moreover, NPWT increases the expression of many cytokines to promote collagen deposition [18]. Furthermore, NPWT in patients with DFUs increases let-7f expression in plasma which may help to control the inflammation and induct the angiogenesis, both associated with wound healing [19]. However, there is a potential problem in the treatment of DFUs by using NPWT. Tissue pressure beneath the foam of NPWT system is increased by the external compression, which could decrease tissue oxygenation in wound beds, especially in diabetic foot. Jung et al. in their study found that NPWT would significantly reduce tissue oxygenation levels in diabetic feet and thus suggested that taking care of the compression of the foam dressing when NPWT should be applied but did not suggest that NPWT should be discarded during the procedure of treating DFUs in consideration of its various positive effects [20].

On the other hand, Liu et al. [4] and Wynn and Freeman [21] both in their review concluded that compared with other wound dressings, it remained unclear whether NPWT could increase the proportion of diabetic foot wounds healed and reduce the time to heal the wounds of DFUs. Likewise, there is no obvious advantage in reduction of wound area by application of NPWT alone in our opinion.

In order to solve the problem, we applied a simple skin stretching system simultaneously in our clinical study. The skin stretching system is an effective method that can accelerate the wound healing by the biomechanical properties of the skin. Compared to traditional surgeries, the technique has the advantages of healing wounds without subsequent reconstruction surgeries, reducing the time for wound closure, and the properties of the stretched skin similar to the adjacent skin [22–25]. There have been various skin stretching devices for wound closure described in the literatures [22, 24, 26–28]. However, the application is limited because of the availability and cost which is particularly important in a developing country with limited resources. In our cases, all materials used in the simple skin stretching system are inexpensive and easily available in most operating theaters. On account of the skin viscoelasticity of diabetic foot being relatively poor, we pay more attention to the assessment of the mobility and quality of the neighboring cutaneous condition before applying the skin stretching system. Several studies in related fields clearly have demonstrated that the skin stretching system is fairly a good adjunctive treatment for diabetic foot wound closure, but better elucidation of the relative indications and contraindications is still needed [29–31].

There are currently few studies about application of a skin stretching system and NPWT simultaneously in diabetic foot wounds. Lee et al. proposed a similar concept in patients with necrotizing fasciitis and showed this method can be an alternative treatment for the necrotizing fasciitis patients with large wounds [32]. Zhang et al. in his study concluded that VSD associated with SSD in patients with stress-induced injuries could improve the therapeutic effect [33]. Wang et al. [34] and Ji et al. [29] reported that the application of skin stretching device and NPWT in diabetic foot ulceration can reduce wound healing time and increase wound healing rate. The examples in these published literatures can support our hypothesis that the combination of simple skin stretching system and NPWT is advantageous.

In our research, the time for closure of complex diabetic wound ranging from 8 to 19 days with a median of 14 days made it more effective than secondary intention healing and skin graft. Furthermore, the procedure was easy to operate, and less time was spent compared with flap transposition. In regard to the absolute rates of reduction, we found a fact that different anatomical sites on the foot had different viscoelastic properties. It was much easier to reduce the area of diabetic foot wound on the side of the foot than on the dorsum of the foot. The reason might be that the soft tissue was more on the side than on the dorsum; thus, the wounds on the side could be treated with greater traction tension to promote wound healing compared with the dorsum.

It was ineluctable that some staples were occasionally dislodged due to constant tension occurring in 2 cases. But it had few influences on wound closure in consideration of the other staples which could still maintain enough traction tension. Additionally, 3 cases of skin edge necrosis occurred in our study. Ji et al. concluded that the skin viscoelasticity and local micro-circulation of diabetic foot was relatively poor [29]. Therefore, we must carefully assess the wounds before using the skin stretch system to avoid the complication of skin necrosis as far as we can. Moreover, pain will occur in the skin stretching system when a persistent force is applied. In the study, all of the patients could tolerate anchorage pain just by taking oral analgesics. The reason might be that the tension of elastic bandage was relatively mild and diabetic peripheral neuropathy affected sensory nerve markedly in lower extremities.

### Limitation

The first limitation is that our study is a retrospective single-center study. To the best of our knowledge, there are few studies to evaluate the value of the skin stretching system and NPWT in the management of complex diabetic foot wounds. It is essential to ensure its safety and validity before a randomized controlled trial is conducted. Second, an important limitation of the simple skin stretching system is the absence of a monitoring system which can adjust the skin tension anytime. Third, the small number of wounds included could be a source of bias. Randomized controlled trials with a larger sample size and a longer follow-up period should be conducted to determine the advantages of the simple skin stretching system and NPWT in future.

## Conclusion

In summary, our results show that the application of the simple skin stretching system and NPWT is an effective and safe approach to complex diabetic foot wounds, which can decrease the time of hospitalization stay and costs, allow earlier rehabilitation and increase patient satisfaction. We believe that this method can provide an alternative choice for surgeons to treat large diabetic foot wounds. Nevertheless, more attentions should be paid to the appropriate patient selection and intraoperative judgment to ensure wound closure and avoid undue complications.

## Data Availability

Not applicable.

## Abbreviations

NPWT: Negative pressure wound therapy
DM: Diabetes mellitus
DFUs: Diabetic foot ulcers
